# Observations on Scurvy, and Its Causes among the U. S. Colored Troops of the 25th Army Corps, during the Spring and Summer of 1865

**Published:** 1866-10

**Authors:** S. Hemenway

**Affiliations:** Late Surgeon 41st U.S. Colored Troops


ARTICLE XXXVI.

### OBSERVATIONS ON SCURVY, AND ITS CAUSES AMONG THE U.S. COLORED TROOPS OF THE 25TH ARMY CORPS, DURING THE SPRING AND SUMMER OF 1865.

By S. HEMENWAY, M.D., late Surgeon 41st U.S. Colored Troops.

During the siege of Richmond, Va., and afterwards in the winter of 1864-5, and the following spring, I had occasion to observe the cause, or rather a combination of causes, to which may be attributed the general appearance of scurvy throughout the 25th Army Corps, upon the arrival of this command in Texas, in June, 1865.

The expedition left City Point, Va., about the 1st of that month. I was informed by Dr. Norton Folsom, Medical Inspector, when he was on an inspecting tour through the Western District of Texas, during the month of September, 1865, that about 60 per cent of the whole strength of the corps had been afflicted with scurvy during the summer. The mortality percentage I do not remember to have heard him say; but a report of sick, during the month of August, 1865, at Edinburg, Texas, shows the number 89, from a command of 1000 men, as having been treated in *hospital;* and from my own personal observations in the hospital during that month, all, or nearly all, had scurvy or some other disease complicated with it. The number of deaths reported was 10, and nearly all the result of a scorbutic condition of the system. So it will be seen, the percentage of mortality, with reference to this part of the command,) which I think can be taken for far better than the average,) was 11 per cent of the whole number of patients treated in hospital. Large numbers were sick from scurvy in *quarters*, and, of course, cannot come under the calculation of those treated in *hospital.* Suffice it to say, that often more than one-half, and sometimes three-fourths, of a regiment were afflicted with scurvy, and were cared for as well as circumstances would permit in *quarters*, in consequence of insufficient hospital accommodations for such large numbers.

This evil had to be remedied, and there were, at the time the colored troops landed in Texas, (in the month of June, 1865,) scarcely any medical supplies, and the quality of the rations for the men *very poor*. As fast as the men were landed at Brazos Santiago, there were, from each U.S. transport, from 50 to 100 of the *worst* cases of scurvy sent to the post-hospital, which was in my charge, and then the only hospital for the whole army of the Rio Grande, having the capacity of 80 beds only. Within one week, there were no less than 500 patients, (chiefly scorbutic,) a few within, and many lying around the hospital buildings, without shelter, in the open air, upon the sand, and supplied with a limited quantity of condensed water, with now and then a supply of fresh *muddy* water from the Rio Grande.

Some 200 or 300 of these patients were immediately sent back to hospitals at New Orleans, but the number of sick again rapidly accumulated upon the arrival of each succeeding transport, and in less than three days the number of patients far exceeded the former. By this time, we had hospital tent-flies up, sufficient to protect several hundred patients in an overcrowded manner. This condition of affairs could not be tolerated for any length of time, and a request was made to have more sick sent away, in order to have room for the number that could be actually accommodated with sufficient space for beds under shelter. I feel justifiable in pronouncing all of these cases scurvy, as I failed to discover, with but very few exceptions, any other disease which was not more or less complicated with a scorbutic condition of the system.

So far as I noticed, the chief characteristic symptoms were a weakened and debilitated state of the system, with emaciation, particularly in those cases accompanied by excessive discharges from the bowels, oftentimes of a dysenteric nature, and sometimes merely a diarrhœa; the gums were very much swollen and projecting over the intervals between the teeth, very similar in size and shape to the ends of one's fingers, and of a dark red or purplish hue, and frequently of a completely black color. These projecting or impending columns of swollen gums very closely approximated each other, and sometimes coalesced, entirely hiding the lateral view of the molar, bicuspid, and canine teeth. Extensive ulcerations of the gums was a universal concomitant, and attended with frequent bleedings. Over various parts of the surface of the body and extremities, there could be detected extensive and diffused ecchymosis, also a dropsical condition of the feet and legs, usually attended with severe pain, sometimes in the joints, simulating rheumatism, and frequently along the continuity of the shafts of the bones of the lower extremities. Vesications were frequently noticeable, from which oozed serum and blood, oftentimes becoming foul ulcers. The muscles of the calf and thigh of the extremities were frequently found to be indurated, and the joints, particularly the knee and ankle, anchylosed.

These patients were universally dejected in spirits, and death ensued after much muscular exertion, or (the patient's appetite being capricious) from hemorrhage, which, in all probability, was induced by excessive and sudden congestion that would inevitably take place during the process of digestion, which must necessarily be more active than is usual during any period of the disease, in consequence of the quantity and quality of the ingesta which the patient happened to be allowed to take. One case, in particular, of the latter kind, I was informed of, as having occurred in one of the wards of the post-hospital at Brazos Santiago. Deaths among scorbutic patients sometimes occurred from pulmonary œdema of passive origin, dependent on the morbid state of the blood.

The *cause* of the appearance of scorbutus among these troops cannot, I think, be attributed *alone* to the short voyage, during the month of June, 1865, from Fortress Monroe, Va., to Brazos Santiago, Texas. There were a few well-marked cases noticed while the troops were encamped near City Point, Va., in May last, before embarking on this expedition. The voyage, however, may have been, and doubtless was, the chief exciting or secondary cause in the developement of scorbutus, dependent on the previous existing causes and the general physical condition of the troops at the time of embarkation. The spaces between decks, into which they were crowded, were commonly dark and illy ventilated, thus constituting the chief exciting or immediate cause.

In one of my weekly reports, in the month of May, when we were encamped near City Point, Va., I reported one case of scorbutus, and stated in the remarks, that I was fearful more cases would shortly be developed. It too frequently happened that in every issue or two of rations, a portion would be found damaged, for example, wormy hard-bread and a damaged condition of beans, rice, and pork. The supply of fresh vegetables was very meagre. The *dessicated* vegetables, these soldiers, as a general thing, disliked very much, and most of them would not partake of the preparation cooked in any manner. Such was the character of the food upon which they subsisted during the previous winter, with the exception of a very bad quality, generally speaking, of fresh beef, issued perhaps once or twice a-week—bad quality, I say, because the beef cattle were not usually in the very best order for beef. It is a well-known fact, how neglected has been the care of beef cattle during their transportation by railroads and steamboats, in not giving them a proper supply of water, or depriving them entirely of it during the whole trip. Of course, the result was a state of exhaustion, and a febrile condition among the cattle was induced, and in this state they were driven off to slaughter-pens, there to be butchered and distributed to the soldiers. Other causes, which may be secondary to this defective mode of subsistence, may be added, such as excessive and long-continued fatigue duty, together with almost constant exposure to damp and cold during the long, wet, winter season. The huts which were built for winter-quarters, were generally very dark, damp, and cold, and the supply of fuel insufficient. Many of these soldiers became despondent, and would not put forth the least effort to make themselves comfortable, but would sit or lie down upon the cold, damp ground, within their huts, without a spark of fire, and most always with their clothes pretty well saturated with water. The water used for drinking purposes was very impure, it being always taken from filthy sloughs, surface-water wells, or some surface-water stream, the washings of old, filthy camp-grounds, etc., etc. Hence, it will be seen, from the statement of these facts, that the cause of the prevalence of scurvy among these troops is very much complicated.

It was remarked by some medical men, after the general appearance of scurvy among the troops in Texas last summer, that they believed that many cases reported as rheumatism, during the winter in front of Richmond, Va., were, in reality, scurvy, of which I have not the least doubt.

When our command landed in Texas, in the month of June, 1865, our means for treatment were very limited for many weeks. The rations issued continued to be of the very worst character, and the water (condensed sea water) poor in quality and small in quantity. As soon, however, as the troops made their way into the interior, they began to improve; and as soon as fresh vegetables were supplied, even in as limited quantities as they were, scurvy began to rapidly decline.

Medicinal agents were tried to some extent. From the use of muriated tincture of iron alone, I have witnessed a gradual improvement in the condition of patients; also from some of the salts of potash—the iodide of potassium in conjunction with chlorate of potash; also topical applications, in the form of lotions, directed to be used for the ulcerated condition of the gums; sulphate of zinc and alumina, but never so rapidly effected as from the juices of fresh vegetables, administered internally.

After the general appearance of scurvy, during the month of June, 1865, it continued to increase rapidly through the following month, after which it began to decline, at first, slowly, and, I think, by the middle of last November, the disease had almost entirely disappeared. This decline of the disease was in consequence of supplies of fresh vegetables furnished, notwithstanding the limited quantities in which they were issued.

A great deal of credit is due to the use of the juice of the plant "*Agave Americana*," indigenous in Mexico, on the border of which country our troops were stationed. A description of the plant is given in the Appendix of the U.S. Dispensatory. I have employed it with some degree of satisfaction, but I cannot say that the good results depended *entirely* upon this remedy, as the scorbutic patients were supplied at times with a few fresh vegetables, such as raw potatoes and onions sliced in vinegar, and pickled cabbage, and thus supplied, the improvement was always, I noticed, much more marked.

